# Magnetic field sensors using arrays of electrospun magnetoelectric Janus nanowires

**DOI:** 10.1038/s41378-018-0038-x

**Published:** 2018-12-03

**Authors:** Matthew J. Bauer, Xiao Wen, Prabal Tiwari, David P. Arnold, Jennifer S. Andrew

**Affiliations:** 1 Dept. of Materials Science and Engineering, University of Florida, Gainesville, FL 32611-6400 USA; 2 Dept. of Electrical and Computer Engineering, University of Florida, Gainesville, FL 32611-6200 USA

## Abstract

The fabrication and characterization of the first magnetoelectric sensors utilizing arrays of Janus magnetoelectric composite nanowires composed of barium titanate and cobalt ferrite are presented. By utilizing magnetoelectric nanowires suspended across electrodes above the substrate, substrate clamping is reduced when compared to layered thin-film architectures; this results in enhanced magnetoelectric coupling. Janus magnetoelectric nanowires are fabricated by sol–gel electrospinning, and their length is controlled through the electrospinning and calcination conditions. Using a directed nanomanufacturing approach, the nanowires are then assembled onto pre-patterned metal electrodes on a silicon substrate using dielectrophoresis. Using this process, functional magnetic field sensors are formed by connecting many nanowires in parallel. The observed magnetic field sensitivity from the parallel array of nanowires is 0.514 ± .027 mV Oe^−1^ at 1 kHz, which translates to a magnetoelectric coefficient of 514 ± 27 mV cm^−1^ Oe^−1^.

## Nanowires: magnetoelectric sensors

A two-phase barium titanate/cobalt ferrite nanowire-based magnetic sensor offers enhanced performance as compared to thin films. Magnetoelectric devices are attractive for magnetic field sensing, and have so far been mostly based on composite thin films of magnetostrictive and piezoelectric phases. However, the film substrate typically limits the size of the magnetoelectric effect, reducing their sensing performance. Now, a team from University of Florida led by Jennifer S. Andrew fabricated composite barium titanate/cobalt ferrite nanowires by electrospinning and suspended them over electrodes, removing the substrate constraints experienced by thin films. Resultantly, their device displays a high magnetoelectric coefficient of 514 ± 27 mV cm^−1 ^ Oe^−1.^ This work demonstrates the potential that one-dimensional materials offer for magnetoelectric based devices including magnetic field sensors.

## Introduction

Magnetoelectrics are unique functional materials in which an applied magnetic field can be used to control an electrical polarization^1^. As such they can offer a lower power alternative to current magnetic field sensors such as Hall Sensors. However to be viable for use in magnetic field sensors, material systems and architectures need to be found which offer high magnetoelectric coefficients (dE/dH). Although single phase magnetoelectrics exist, they are comparatively rare^2^. On the other hand, composite magnetoelectrics are capable of producing greater magnetoelectric effects. For magnetoelectrics the figure of merit is the magnetoelectric coefficient (α_V_), which is quantified as the magnitude of the electric field (dE) generated in a material in response to an applied magnetic field (dH), α_V_ = dE/dH^1^. Composite magnetoelectrics are typically composed of magnetostrictive and piezoelectric phases which share an interface. When exposed to an applied magnetic field the magnetostrictive phase undergoes a shape change, which imparts a strain to the piezoelectric phase, thereby inducing an electrical polarization. When magnetoelectric composites are fabricated as thin films, the strain transfer between the magnetostrictive and piezoelectric phases is typically limited by the underlying substrate leading to a reduction in the magnetoelectric effect^1,3–5^. Less rigidly clamped 1-D magnetoelectric nanostructures could offer increased magnetoelectric coefficients. Enhancements of up to a few orders of magnitude seem feasible based on theoretical and scanning probe microscopy measurements^6^.

However, to make use of magnetoelectric nanowires demands nanomanufacturing processes that enable (a) the synthesis of nanowires with controlled length, (b) the ability to direct the assembly of these nanowires into ordered arrangements while avoiding substrate clamping effects, and (c) the ability to make suitable electrical connections to one or more nanowires.

**Fig. 1:**
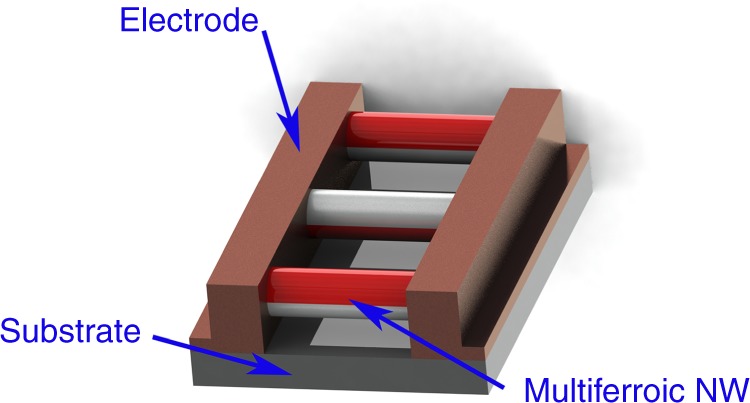
A device schematic showing magnetoelectric barium titanate–cobalt ferrite nanowires assembled in parallel across metal electrodes

Here, we seek both to demonstrate suitable nanomanufacturing methods and to confirm the increased magnetoelectric coefficients 1-D structures offer by fabricating magnetic field sensors consisting of an array of magnetoelectric biphasic fibers suspended across electrodes (Fig. 1). Specifically we selected the barium titanate and cobalt ferrite system for our 1-D magnetoelectric, as it has previously been shown to have a significant magnetoelectric effect in bulk^7–12^ and thin film form^13^. We chose a bilayer, Janus, morphology in order to promote the bending mode in the magnetoelectric, which will likely allow for greater strain in the nanowire while suspended between the electrodes.

Several methods exist to fabricate 1-D magnetoelectrics including sol-gel electrospinning^14–18^, hydrothermal synthesis^19^, and various chemical and physical vapor deposition processes^20^. We opted for sol-gel electrospinning as it has been previously shown to be capable of producing magnetoelectrics with a wide range of compositions and various connectivities including fibers with Janus^16–18^, core shell^15,21^, and randomly dispersed morphologies^14^. Sol−gel electrospinning was also chosen due to its scalability and it’s relatively low cost. Additionally, when combined with a bottom up assembly technique, the high temperature calcination step can be performed off substrate making it CMOS compatible and feasible for application in a manufacturing setting^22,23^.

Sol-gel electrospinning is a method by which a ceramic/polymer solution is drawn, often from a syringe needle, into a nanofiber using a large electric field that is applied between the solution and a counter electrode^24,25^. While the sol-gel solution is extruded into a droplet at the tip of the syringe needle, a surface charge forms on the droplet due to the applied voltage. As the solution accumulates a surface charge it is pulled toward the counter-electrode by the electric field in a shape referred to as a Taylor cone^24,25^. When the surface charge on the solution overcomes surface tension, a charged jet is emitted from the Taylor cone. This jet is then accelerated toward the counter-electrode by the applied electric field, during which time the solvent evaporates and hydrolysis and condensation of the precursors occurs^24,25^. After electrospinning, the as-spun amorphous fibers undergo a high temperature calcination step to burn off the polymer and to crystallize the ceramics.

For the assembly of 1-D magnetoelectrics into devices, it is desirable to have discrete nanowires. Therefore, we developed a controlled salt calcination method, where an increased temperature ramp rate was employed to break up the as-electrospun continuous nanofibers into shorter nanowires and the salt prevented agglomeration during calcination, resulting in the formation of discrete nanowires^26^. This is similar to a method that we previously reported for the fabrication of barium titanate nanowires that leveraged the tension caused by the shrinkage of the as-spun fibers during the calcination step, while clamped by carbon tape, to break up the nanowires radially^27^. For assembly a nanowire slightly longer than the electrode gap is desirable since the nanowire must bridge the electrode gap for electrical connections to be made and nanowires which are too long may quickly settle out of solution (Fig. 1). As such we sought to find electrospinning and calcination parameters which could provide control of the nanowire lengths. The two parameters we focused on in this paper are the electrospinning voltage, as a means to control the as-spun fiber diameter and calcination ramp rate. Though these are not the only parameters which could control nanowire length, they can be readily applied to other systems.

One of the challenges in producing devices out of nanomaterials, is assembling them in such a way that maintains their structure and properties. Thus, we chose to utilize AC electrical assembly as it is well suited to assembly of nanowires/particles. Although various types of bottom up fabrication techniques exist, including electrophoretic deposition^28^ and 3D printing methods^29^, AC electrical assembly utilizing the dielectrophoretic effect is particularly suited to producing arrays of nanowires suspended across electrodes (Fig. 1) and its scalability has been previously demonstrated to produce dense arrays of nanowire^30,31^. In AC electrical assembly a nanowire, or particle suspended in solution forms a dipole in response to an applied electric field, and experiences a force along the gradient of the electric field, toward the electrode gap, referred to as the dielectrophoretic force^30,32^. Once near the electrode gap short range capacitive forces act to orient the nanowires across the gaps^30^. Other forces present include dipole-dipole interactions, electrostatics, capillary forces, and AC-electroosmosis^30,32^. These can cause repulsion or chaining between nearby nanowires, adhesion to the substrate, disruption of nanowires upon drying, and a flow of solvent around the nanowires, respectively, to varying extents depending on the assembly parameters, such as the electrical and rheological properties of the nanowires and solvent. Thus, these assembly parameters can be tuned to achieve improved nanowire assembly.

As AC electrical assembly is highly dependent on the electrical properties, namely conductivity and permittivity, of the solvent and particles used, we attempted electrical assembly of the Janus nanowires in various solvents: water, ethanol, 2-methoxyethanol, and butanol. The motivation for this investigation was to obtain improved assembly guided by the real portion of the Clausius Mossotti factor for a particle in solution at high frequencies^33^.

1 $${\it{Re}}\left[ K \right] \approx \left( {\varepsilon _{particle} - \varepsilon _{medium}} \right)/\left( {\varepsilon _{particle} + 2\varepsilon _{medium}} \right)$$

with *Re*[*K*] > 0 giving the desired positive dielectrophoretic force, an attraction of the nanowires toward the electric field gradient maxima i.e., the edges of the electrodes. Post assembly we created upper electrical contacts using lithography, sputter coating and electroplating.

For characterization of the magnetoelectric nanowires and assembled arrays we aimed to verify ferrimagnetism in the cobalt ferrite phase^34^, ferroelectricity in the barium titanate phase, and finally quantify the magnetoelectric coefficient; this was accomplished through vibrating sample magnetometry (VSM), capacitance voltage (C–V) measurements, and direct magnetoelectric measurements respectively. A direct magnetoelectric measurement  that records an electrical response to an applied magnetic field^35,36^, were focused on as our main characterization technique for magnetoelectricity as this is the mechanism by which the array can be used for passive magnetic field sensing.

In this manuscript we detail the fabrication of the first passive magnetic field sensors using 1-D magnetoelectric nanostructures through scalable nanomanufacturing methods. The presented fabrication techniques are readily applicable to a wide range of magnetoelectric systems allowing their extension to many magnetoelectric-based devices.

## Results

### Nanowire fabrication

**Fig. 2:**
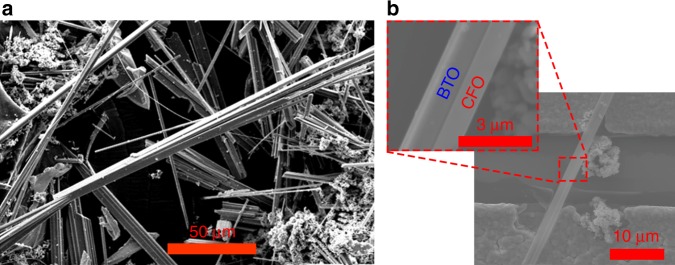
**a** Scanning electron micrograph of Janus barium titanate - cobalt ferrite (BTO–CFO) nanowires post salt calcination. These nanowires were electrospun at 2 kV cm^−1^ and calcined for 8 h at 1100 °C with a ramp rate of 10 °C min^−1^. **b** Scanning electron micrograph of a single Janus BTO–CFO nanowire post assembly across the nanowire arrays, the two phases of the Janus nanowire are labelled here as BTO and CFO solely for illustrative purposes as in the micrograph it is uncertain which section of the nanowire corresponds to each phase

**Fig. 3:**
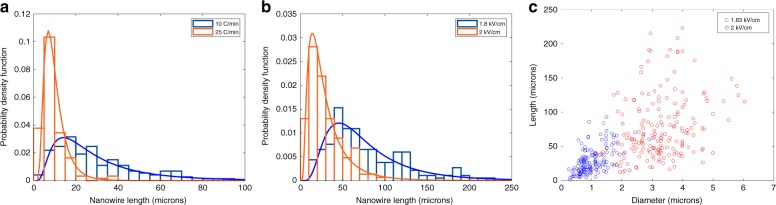
**a** Histogram and fitted lognormal distributions for nanowires calcined at 25 and 10 °C min^−1^ with an electrospinning voltage of 2 kV cm^−1^ showing a decreasing nanowire length with increasing calcination temperature. **b** Histogram and fitted lognormal distributions for nanowires electrospun at 2 and 1.83 kV cm^−1^ with a constant calcination ramp rate of 10 °C min^−1^ showing an increasing nanowire length with decreasing electrospinning voltage. **c** Nanowire lengths and voltages from nanowires electrospun at 2 and 1.83 kV cm^−1^ with a constant calcination ramp rate of 10 °C min^−1^ showing a positive correlation between nanowire diameter and length

We formed Janus barium titanate and cobalt ferrite magnetoelectric nanofibers via sol gel electrospinning and utilized a rapid calcination ramp rate to shrink the fibers radially, creating tension to break them into shorter nanowires. A scanning electron microscope image of calcined nanowires is shown in Fig. 2a, revealing that the electrospinning and subsequent calcinations utilizing fast ramp rates were successful in producing barium titanate/cobalt ferrite Janus nanowires. An image of a single Janus nanowire is shown in Fig. 2b as here it is easier to distinguish the two distinct halves of the wire. We investigated the effects of electrospinning voltage and calcination ramp rate on nanowire length, as for the subsequent electrical assembly step we desire nanowires sufficiently long to span the electrode gap but not so large as to settle quickly out of solution. We believed that increasing the calcination ramp rate could decrease nanowire length as the resultant faster polymer burnoff should lead to the breakup of the fibers into shorter nanowires. This is supported by Fig. 3a which shows a decrease in nanowire lengths from 29.06 ± 19.34 μm to 19.34 ± 6.08 μm when the calcination ramp rate was increased from 10 to 25 °C min^−1^. We also hypothesized that as-spun nanofibers with larger diameters would in turn produce longer nanowires. The electrospinning voltage can be readily tuned to control the fiber diameter, where higher electrospinning voltages result in smaller diameter fibers. This is because an increase in applied field produces a larger elongating force on the fiber jet during electrospinning, leading to smaller diameter nanofibers, which given the same calcination ramp rate would form similar aspect ratio, and thus shorter nanowires. Figures 3b, c demonstrate that a decrease in electrospinning field from 2 to 1.83 kV cm^−1^ resulted in longer nanowires, increasing the length from 29.06 ± 19.34 μm with 2 kV cm^−1^ to 77.43 ± 46.11 μm with 1.83 kV cm^−1^, and larger diameter fibers with similar aspect ratios. This is also supported by the positive correlation coefficient between nanowire length and diameter as shown in Fig. 3c of *R* = 0.604 with a p-value of *p* < 0.01 (>99% confidence level). It is also important to note the heteroskedasticity in the Fig. 3c; i.e., that an increasing nanowire diameter is positively correlated with increased nanowire length, but also increased length variation.

**Fig. 4:**
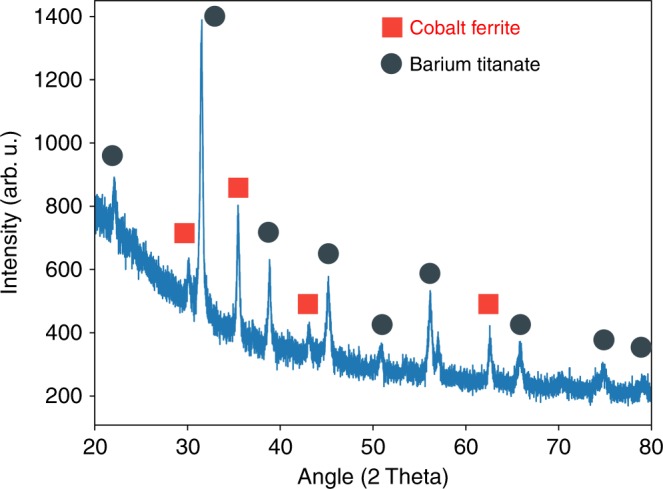
X-ray diffraction spectra of the Janus nanowires post calcination showing peaks indicative of tetragonal barium titanate and spinel cobalt ferrite

**Fig. 5:**
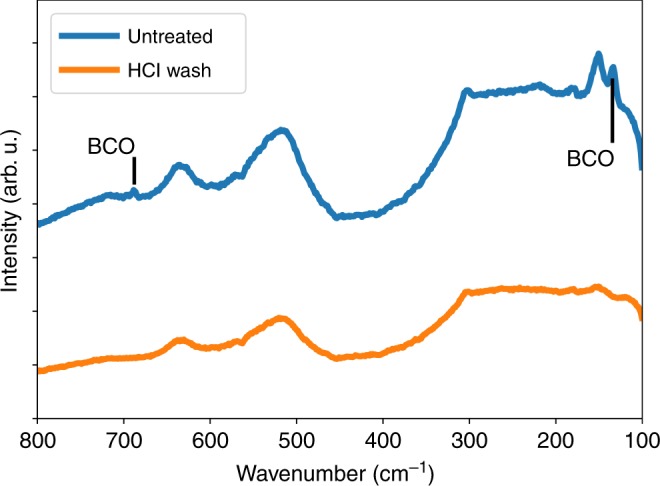
Raman spectra of single phase barium titanate nanowires used to test whether barium carbonate (BCO) can be successfully removed with a dilute hydrochloric acid (HCl) wash. Absence of the barium carbonate peaks in the as HCl treated sampled demonstrates that this wash was successful in removing BCO impurities

To verify the crystal structure of the calcined barium titanate and cobalt ferrite nanowires we first performed X-ray diffraction (Fig. 4), then subsequently analyzed the results using Rietveld refinement. From this we found the fibers to be comprised of 62 wt.% tetragonal barium titanate, P4mm, and 38 wt.% spinel cobalt ferrite, Fd-3m. The agreement indices of the refinement, *R*_expected_ = 5.19, *R*_weighted_ = 5.49, and *χ*^2^ = 1.26, indicate that these are indeed the structures present and that there are low levels of or no crystalline impurities. Though XRD showed no impurities, we have seen surface a barium carbonate surface impurities in as-calcined barium titanate nanowires formed via sol gel electrospinning^33^. We tested the removal of this impurity via acid treatment with dilute HCl in single phase barium titanate nanowires using Raman spectroscopy; we used single phase wires for this test so that any signal from the cobalt ferrite phase would not obscure the barium carbonate peaks. In Fig. 5 we see that the barium titanate phase contains barium carbonate peaks which are no longer present after acid treatment, showing that a dilute HCl treatment can remove the barium carbonate impurity from the as-calcined wires.

### AC electrical assembly

**Fig. 6:**
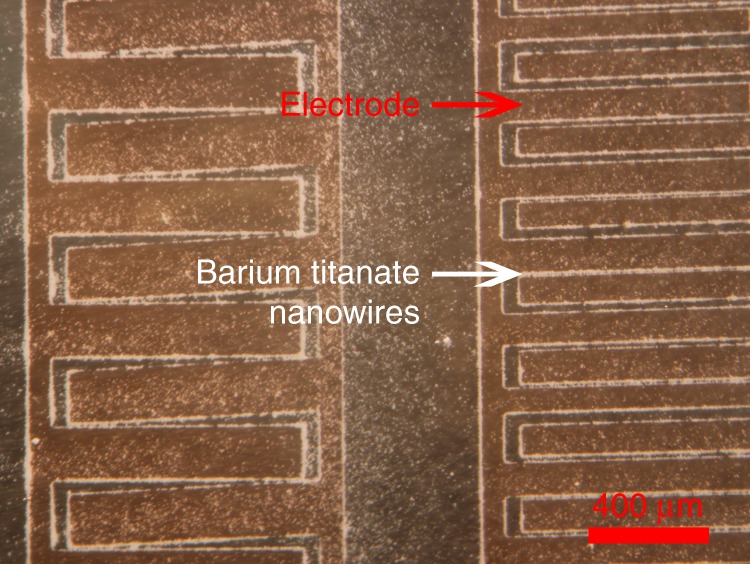
Successful assembly of barium titanate nanofibers in water, post barium carbonate removal with a dilute HCl wash and suspension using citric acid and adjusting the pH to around 9, at 5 kHz and 20 Vpp

**Fig. 7:**
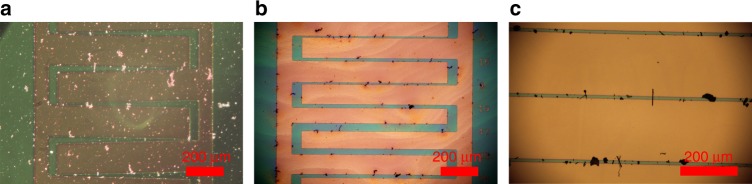
Assembly of Janus nanofibers in **a** ethanol, **b** 2-methoxyethanol, and **c** butanol at 5 kHz and 20 Vpp. While positive dielectrophoresis was observed with all three solvents, ethanol evaporated quickly leaving less time for assembly and butanol appeared to produce slightly better assembly than 2-methoxyethanol

**Fig. 8:**
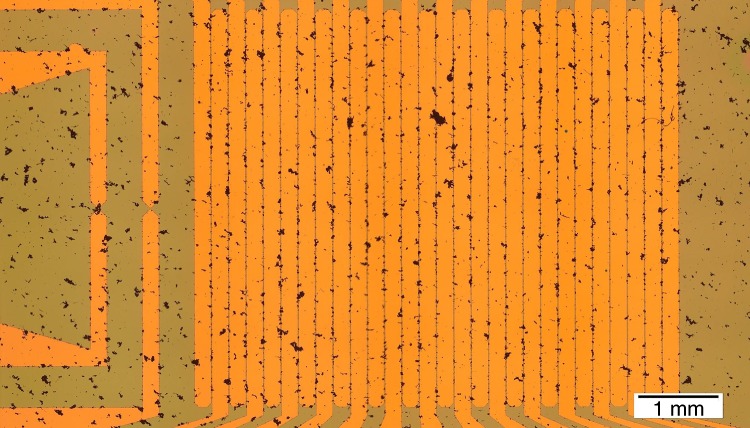
Assembly of Janus nanowires in butanol at 5 kHz and 42 Vpp in the test array with a linear density of 19 NWs mm^−1^

**Fig. 9:**
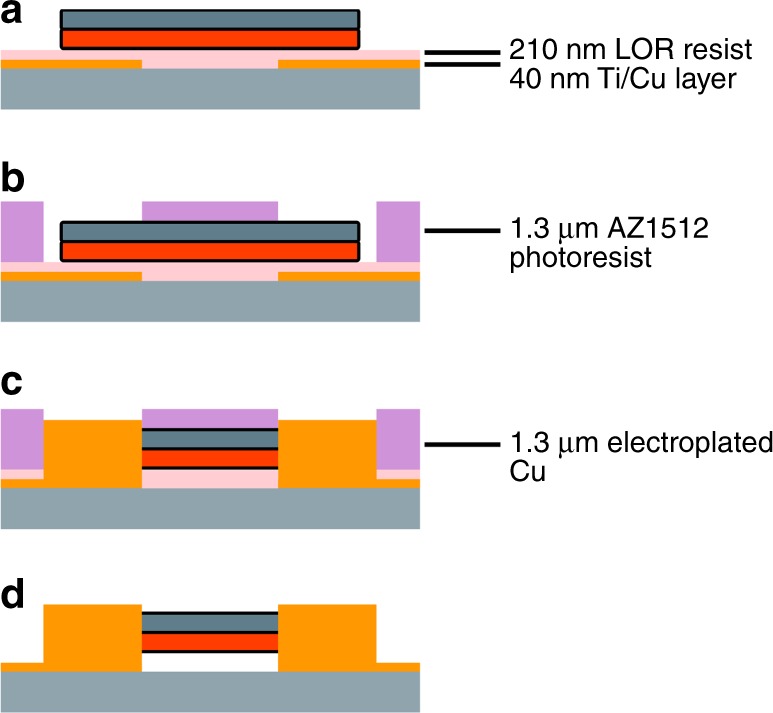
Formation of upper electrical contacts across the nanowires via **a** patterning of Ti/Cu electrode patterns via sputtering and lift-off; followed by spin coating blanket layer of LOR resist; and  assembly of nanowires; **b** spin coating and patterning AZ1512 photoresist to expose ends of the nanowires; **c** electroplating copper to make electrical contacts with nanowire; and **d** stripping of the remaining photoresist

To assemble the magnetoelectric Janus nanowires across parallel electrodes to form our devices we dispersed them in solution and utilized an AC electrical assembly technique^30^. To achieve successful electrical assembly, a number of electrical assembly parameters including nanowire surface treatments, solvent, and frequency of the applied electric field were varied. Initial assembly attempts of Janus nanowires in water were unsuccessful, which we attributed to the presence of low dielectric constant barium carbonate on the surface of the nanowires. While the removal of barium carbonate promoted strong positive dielectrophoresis in single phase barium titanate nanowires in water, Fig. 6, it did not sufficiently improve assembly in the Janus nanowires. This suggests that the relatively low permittivity of cobalt ferrite is decreasing the dielectrophoretic force to a large extent. Thus, we had sought to decrease the permittivity of the solvent relative to the nanowires to promote assembly, performing assembly in ethanol, 2-methoxyethanol, and butanol. Assembly in each of these solvents showed good positive dielectrophoresis (Fig. 7), however the ethanol evaporated rather rapidly, not allowing much time for assembly to occur. As such, butanol was selected for the subsequent assemblies which led to good assembly in the test arrays which allowed individual nanowire rows to be measured (Fig. 8). In an attempt to optimize the frequency for electrical assembly we swept the frequency from 100 Hz–10 MHz while observing the nanowires in solution. It appeared that a frequency of around 5 kHz performed well for the nanowires in the lower dielectric constant solvents, hence this frequency was used for assembly of the nanowires across the electrodes in the test array. Figure 8 shows successfully assembled nanowires across the test electrodes using 42 volts peak to peak @ 5 kHz in butanol post barium carbonate removal. We found the linear density of the as assembled nanowires to be approximately 19 NWs/mm across 27 rows of nanowires. After assembly we formed upper electrical contacts with the nanowires via lithography and deposition of upper electrical contacts via copper electroplating as shown in Fig. 9.

The resultant linear density of the nanowire assembly post electrode deposition decreased to 3.6 NWs/mm. During deposition of the upper electrical contacts, this decrease in nanowire density can be attributed to the fact that some nanowires were not sufficiently long enough to be covered by the upper contacts, and loss of adhesion in the upper electrodes. While this density was sufficient to test the concept of using these nanowires to form a passive magnetoelectric magnetic field sensors and should allow for miniaturization, methods to produce a higher density of assembly will be the focus on ongoing research, to allow fabrication of devices with even smaller footprints. A higher density assembly could likely be achieved through the use of alternative solvents or further tuning of the assembly frequency, or the implementation of a microfluidic channel^37^. To increase the proportion of nanowires which remain assembled across the electrodes through the process of depositing upper electrical contacts the proportion of the nanowires long enough to be covered by the electrodes could be increased. To do so the electrospinning parameter space could be further explored to increase the average length of the electrospun nanowires while maintaining a low variance in their distribution. To help adhesion of the upper contacts the surface of the wafer could be cleaned via carbon dioxide snow cleaning^38^ after photolithography and before electrode deposition, between steps 2 and 3 in Fig. 9.

### Characterization

**Fig. 10:**
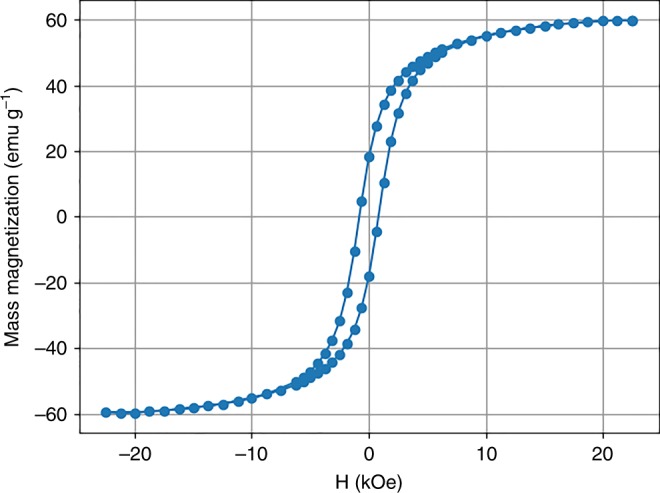
Mass magnetization curve indicating ferrimagnetism in the cobalt ferrite phase of unassembled Janus nanowires from the same batch as those used to fabricate the nanowire array

**Fig. 11:**
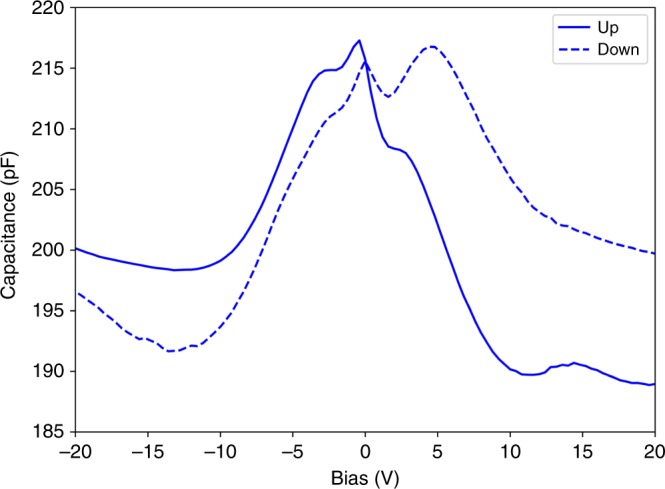
Capacitance–voltage (C–V) measurement from the Janus nanowire array demonstrating ferroelectricity in the barium titanate phase. The test signal level was set to 100 mV at 750 kHz

A sample of calcined nanowires (not assembled, but from the same batch used for the assembly process) was measured by a VSM to confirm a ferrimagnetic response. Figure 10 shows the hysteresis loop for the randomly oriented nanowires, showing a saturation of 60 emu/g, a remanence of ~18 emu/g, and a coercivity of ~0.8 kOe. Figure 11 shows the C–V curves for an assembled row of nanowires. The data for the nanowires shows a hysteretic behavior for the up sweep and down sweep, confirming both electrical connectivity and a ferroelectric response due to the barium titanate in the wires. For comparison the measured C–V curve for an empty substrate did not exhibit a ferroelectric response.

Magnetoelectric measurements were performed (a) using the lock-in technique^36^ with a DC bias field up to 8 kOe provided by an electromagnet and (b) using only an AC field (zero DC bias) provided by a Helmholtz coil pair in conjunction with a rotational measurement setup to explore and quantify the effect of electromagnetic induction. In the rotational setup the nanowire array and electrical connections could be rotated 360° within a set of Helmholtz coils to find the angle of no induction, which occurs when the loop of wire formed by the nanowire array and electrical connections is orthogonal to the applied field.

**Fig. 12:**
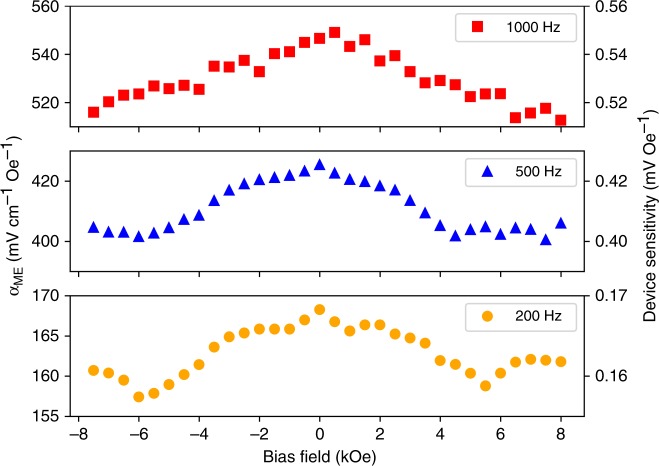
Plot of the magnetoelectric coefficients of a row in the Janus nanowire array measured using the lock-in technique as a function of magnetic bias field at 200, 500, and 1000 Hz

The magnetoelectric coefficients of a row of nanowires in the nanowire array were measured by the lock-in technique^36^, shown in Fig. 12, as a function of bias field at 200, 500, and 1000 Hz. Here, for all three frequencies, a zero bias magnetoelectric effect is observed. Where previously observed, this effect has been attributed to remanent magnetization in the magnetic phase^39–42^, which is consistent with the remanence observed in the magnetic hysteresis loops of the nanowires. This phenomenon can be thought of as a “self-biasing” effect^43–46^, in that the remanent magnetization of the wires creates an internal demagnetizing field that provides a bias field to the magnetic phase.

Figure 12 also reveals a general increase in magnetoelectric coefficient with frequency, which has been previously reported and can be attributed to changes in the dielectric constant of the constituent phases as a function of frequency^39,47^. This likely leads to a reduction in charge leakage through the magnetostrictive cobalt ferrite phase with increasing frequency.

**Fig. 13:**
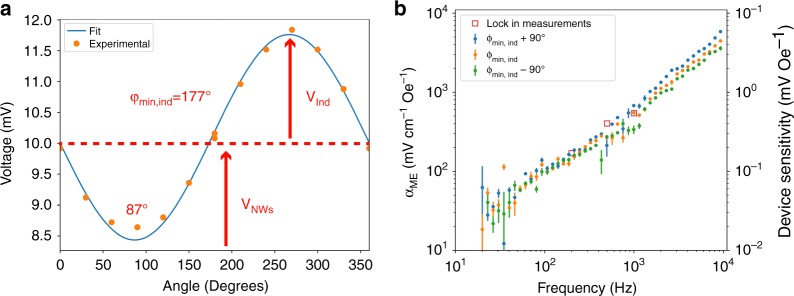
**a** Effect of angle of the Janus nanowire array angle on the observed voltage generated by the array with a 4.9 Oe applied AC field at 1 kHz, as well as the fitted sine function used to find the angle of zero induction *φ*_0_. **b** Measured magnetoelectric coefficients as a function of frequency from the nanowire arrays at angles of approximately no inductive effects, *φ*_min,ind_ = 177°, the angle of maximum constructive induction, *φ*_min,ind_ + 90°, and maximum destructive induction, *φ*_min,ind_ + 90°

To obtain additional magnetoelectric measurements at zero-bias, a rotational measurement setup to eliminate inductive effects was constructed and the zero-bias nanowire array response was directly measured by an oscilloscope. This setup allows for the angle of the array with respect to the applied magnetic field to be adjusted systematically in order to explore the influence of electromagnetic induction in the measurement. Specifically, we used this setup to (1) find the angle at which the normal of the array and electrical connections were orthogonal to the applied magnetic field, eliminating induction, and perform magnetoelectric measurements at this angle (2) measure the effective magnetoelectric coefficients at ±90° from this angle, where induction either maximally constructively or destructively interfered with the voltage generated through the magnetoelectric effect. Figure 13a shows the the voltage output of the nanowire array as a function of the angle of the nanowire array with respect to a 1 kHz applied AC field. Here, it can be seen that there is an inductive contribution which is angle dependent and an angle independent magnetoelectric response. As expected the inductive contribution is sinusoidal and can be fit to find the angle of zero inductive contribution φ_min,ind_ = 177°. The results of direct magnetoelectric measurement spectra can be seen in Fig. 12b which compare the effective magnetoelectric coefficients as measured at *φ*_min,ind_, an angle with induction additive to the magnetoelectric effect *φ*_min,ind_ + 90° and an angle at which induction which opposes the magnetoelectric effect *φ*_min,ind_ −90°. It is observed that the maximum inductive contribution of the nanowire array is much smaller than the magnetoelectric effect, 15% when rotated to an angle at which the maximum inductive contribution occurred at 1 kHz.

Figure 13b shows the measured device response from 20 Hz to 10 kHz. Again, a general increase in magnetoelectric effect with respect to increasing frequency is found, and there is good agreement with the lock-in measurements (denoted by the red squares). For the case of minimal induction, magnetoelectric coefficients with zero bias at 200 Hz, 500 Hz, and 1 kHz were found to increase from 152 ± 13 to 267 ± 14 to 514 ± 27 mV cm^−1^ Oe^−1^. These values correspond to device sensitivities of 0.152 ± 0.013 to 0.267 ± 0.014 to 0.514 ± 0.027 mV Oe^−1^, at 200 Hz, 500 Hz, and 1 kHz, respectively.

## Discussion

After completing the magnetoelectric measurements we compared the magnetoelectric coefficients from the nanowire rows in our arrays from those found in the literature for the barium titanate/cobalt ferrite system in thin film^13^ and bulk^7^ structures. Here we found that the nanowires exhibit significantly larger magnetoelectric coefficients (514 ± 27 mV cm^−1^ Oe^−1^ at 1 kHz) compared to values previously observed with this material system. For example, bulk barium titanate / cobalt ferrite systems have been shown to demonstrate magnetoelectric coefficients of 50 mV cm^−1^ Oe^−1^ at 60 kHz^7^ Zhang et al. reported 104 mV cm^−1^ Oe^−1^ at 1 kHz for BTO-CFO single crystal thin films^13^. The increased magnetoelectric coefficient observed in the nanowire arrays in comparison to thin films seems reasonable based on theoretical study of substrate clamping effects^48^ and PFM measurements made by Xie et al. on PZT-CFO fibers^6^ and a comparison to PZT-CFO thin films^39^.

In conclusion, we fabricated a magnetic field sensor via the assembly of arrays of magnetoelectric nanowires using methods that are readily scalable, economical, and CMOS compatible. We demonstrated that magnetoelectric nanowires with controllable lengths can be prepared by tuning both the electrospinning and calcination conditions and that dielectrophoretic assembly methods allow the fabrication of functional arrays of magnetoelectric nanowires. We measured the direct magnetoelectric effect from the nanowire array using the lock-in technique at 200, 500, and 1000 Hz with bias fields between −8 and 8 kOe. We also measured the magnetoelectric coefficients of the assembled nanowire arrays at zero-bias from 20 Hz to 10 kHz and observed magnetoelectric coefficients notably greater than bulk and thin film systems comprised of the same magnetoelectric material.

Additional improvements can be made to the electrospinning, electrical assembly, and upper electrode formation parameters to maintain reliable device fabrication in increasingly smaller electrode footprints, allowing even smaller devices to be manufactured. Furthermore, as all processing steps on the wafer were designed to be performed at low temperature these devices can be readily integrated with on chip signal processing components with the potential to further reduce device size.

## Materials and methods

### Nanowire fabrication

First barium titanate and cobalt ferrite sol gel precursor solutions were prepared. A barium titanate ceramic solution was prepared by dissolving 0.4246 g barium acetate in 3 ml acetic acid at 80 °C under constant stirring, followed by cooling to room temperature. After 1 h 0.493 ml of titanium isopropoxide was added. Simultaneously, a polymer solution was prepared by dissolving 0.4 g polyvinylpyrrolidone in 3 ml ethanol under constant stirring. After an additional hour, the ceramic solution was added dropwise to the polymer solution under constant stirring. Similarly a cobalt ferrite ceramic solution was prepared by dissolving 0.484 g cobalt nitrate hexahydrate and 1.342 g ferric nitrate nonahydrate in 2 ml of acetic acid and 0.75 ml ethanol. After stirring for 1 h 0.412 ml acetylacetone was added. Simultaneously a polymer solution was prepared by dissolving 0.4 g polyvinylpyrrolidone in 3 ml ethanol. After an additional hour the ceramic solution was added dropwise to the polymer solution under constant stirring.

Both solutions were co-electrospun side by side to form Janus nanofibers, with one semi-cylinder of the fiber consisting of cobalt ferrite and the other barium titanate^16^. The ceramic/polymer nanofibers were then calcined; burning off the polymer and breaking them along their length, and crystallizing the amorphous as-spun ceramic at 1100 °C. To prevent sintering together of nearby nanowires a salt calcination was performed in sodium chloride. The effect of electrospinning voltage and calcination ramp rate on fiber diameter were studied. After salt calcination the salt and nanowires were immersed in a beaker of water, wherein the salt dissolved. After which a permanent magnet was used to attract the nanowires to the bottom of the beaker and the excess water was decanted. The nanowires were placed in dialysis tubing, and dialyzed in deionized water to remove the salt. Post calcination the nanowires were imaged via scanning electron microscopy, and their crystal structure was analyzed via X-ray diffraction with Rietveld Refinement. A dilute HCl wash was used to remove any barium carbonate from the surface of nanowires, verification of the effectiveness of this technique, as tested on barium titanate, was obtained via Raman spectroscopy.

### AC electrical assembly

Electrical assembly of the Janus nanowires was attempted in water, ethanol, 2-methoxyethanol, and butanol. For assembly in water, citric acid was added to the solution such that the pH of the solution was around 9 to achieve a more stable suspension of the nanowires. For the ethanol, 2-methoxyethanol, and butanol solutions the nanowires were placed a centrifuge tube and nearly all of the water was similarly decanted while the nanowires were held in place with a permanent magnet. The nanowires were then dried in a vacuum oven, the respective solvent was added, and the solvent/nanowire solution was sonicated and vortexed.

During the electrical assembly a droplet of the nanowire solvent solution was placed over the electrode array. For the initial assemblies of nanowires in parallel across interdigitated electrodes to tune assembly parameters, a function generator was used which could supply a sinusoidal voltage of 20 volts peak to peak (Vpp), and the the frequencies were swept from 100 Hz–10 MHz. Since 5 kHz seemed to promote positive dielectrophoresis, this frequency was used with an applied voltage of 20 Vpp across the electrode gaps, to compare the assemblies using each of the solvents. Once a suitable solvent for assembly was found, test arrays were fabricated which allowed multiple rows of nanowires to be measured from a single assembly. When performing assembly on these test arrays a pulse generator which was capable of producing higher voltages was implemented, and these assemblies were performed with 42 Vpp at 5 kHz. After nanowire assembly, upper electrode contacts were formed with the nanowires via spin coating AZ1512 photoresist, optical lithography and removal of photoresist from the ends of the nanowires, electroplating copper, and stripping of the remaining photoresist.

### Characterization

To verify ferrimagnetism in the barium titanate/cobalt ferrite nanowires, the mass magnetization curve, M–H, was obtained using a VSM. For the M-H curve the field was swept from 22 to −22 kOe, then swept back up to 22 kOe. This M–H curve was not obtained from the assembled nanowires on the array but in nanowires fabricated in the same batch as those assembled.

To verify ferroelectricity in the barium titanate / cobalt ferrite nanowires, C–V curves were measured using a Hewlett Packard 4294 A Impedance Analyzer by probing the response of an assembled row of nanowires. The C-V bias voltage was swept from −30 to +30 V and from −30 to +30 V with an AC source voltage of 100 mV and frequency of 750 kHz. A low AC source voltage was desired for the nanowire C-V curves so that the AC signal would have a minimal effect on the polarization state of the ferroelectric barium titanate. For comparison, an empty electrode substrate (no nanowires) was measured with the same AC source voltage.

**Fig. 14:**
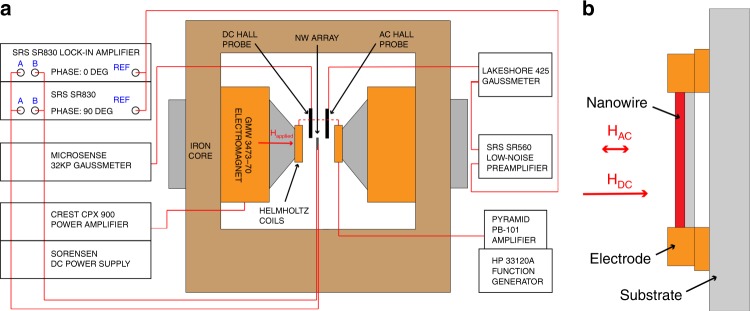
**a** Schematic diagram of the lock-in magnetoelectric measurement setup. Here a large DC bias H-field can be applied to the nanowire arrays alongside an AC H-field. Lock-in amplifiers, phase-locked to the magnetic field signal are used to measure the voltage from the nanowire array. The low noise preamplifier was used to amplify the Hall probe signal to help maintain phase lock to the field. **b** Schematic of the nanowire array showing the direction of the applied AC and DC fields in relation to the nanowire axis

A lock-in measurement setup^36^, depicted in Fig. 14a, was used to measure the magnetoelectric effect in the nanowire arrays as a function of bias field and frequency. As shown in Fig. 14b, the magnetic fields were applied transverse to the long axis of the nanowires. The DC bias field H_DC_ was cycled from 0 kOe up to 8 kOe, then from 0 kOe down to -8 kOe using a GMW 3473-70 electromagnet with a 5 second dwell at each bias field value. The DC bias field was measured using a Hall probe and Microsense Gaussmeter, which also provided closed-loop control of the current delivered to the electromagnet. A small AC field at 200, 500, or 1000 Hz was applied via Helmholtz coils placed at the electromagnet poles. The Helmholtz coils were powered using a Hewlett Packard 33120 A function generator and a Pyramid PB-101 amplifier. For each frequency a 1 Oe AC field amplitude was targeted, but the actual AC field amplitude varied with the DC bias field by ~20% due to changes in the reluctance of the electromagnet. Consequently, the actual applied AC field was measured by a second Hall probe connected to a Lakeshore 425 Gaussmeter for use in calculating the magnetoelectric coefficient. The analog output of the AC field signal from the Gaussmeter was amplified by a Stanford Research Systems SR560 low-noise preamplifier and served as the phase reference for the lock-in measurements. The voltage from the nanowire array was measured using two Stanford Research Systems SRS830 lock-in amplifiers phase locked at 0 and 90 degrees relative to the AC magnetic field.

The voltage waveform produced by the nanowire array is potentially a combination of the magnetoelectric response as well as an electromagnetic induction. For a sinusoidal excitation, these two constituent signals would each be sinusoids at the same frequency as the applied AC magnetic field, but they would be expected to have differing phases (relative to the AC magnetic field). The total measured signal can thus be expressed in phasor form as $\tilde V_{MEAS} = \tilde V_{ME} + \tilde V_{IND}$, where a tilde over a variable is used to indicate that it is a phasor. Consequently, efforts were made in the experimental setup to minimize the induction signal by orienting the wires connecting to the nanowire array in a manner so as to minimize the cross-sectional area of any loop. As will be shown in a second experiment, the induction voltage is relatively small (<15%) compared to the magnetoelectric signal. The magnetoelectric coefficient was calculated as

$$\alpha _V = \frac{{\left| {\tilde V_{ME}} \right|}}{{L \cdot \left| {H_{AC}} \right|}},where\,L = 10\,\mu m$$

**Fig. 15:**
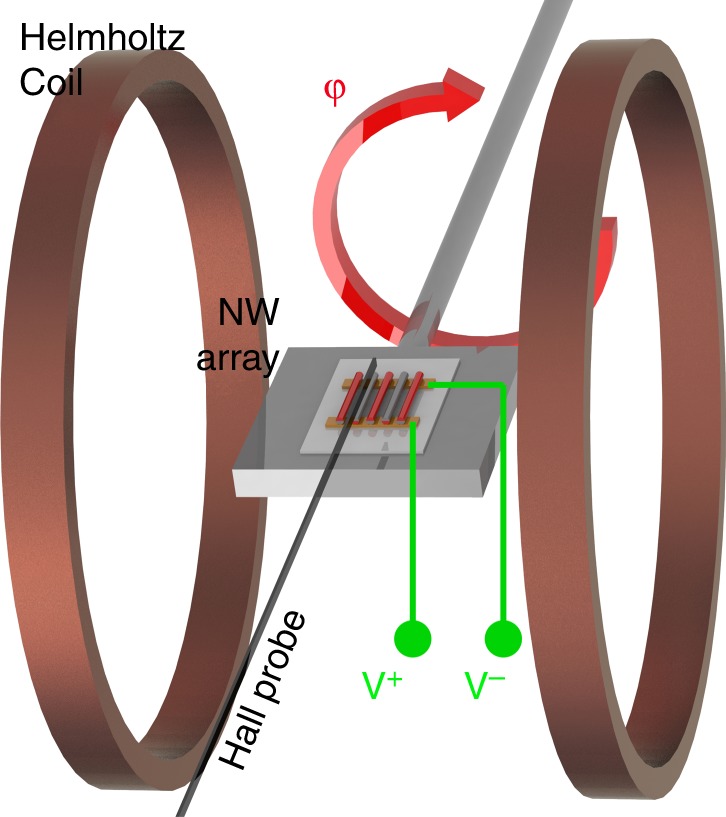
Schematic of the rotating magnetoelectric measurement setup, where the angle of the array with respect to the applied magnetic field can be adjusted to explore the effects of induction on the measured magnetoelectric coefficient

The nanowires generate sufficiently large voltage to allow direct measurement of the output voltage using an oscilloscope. To obtain additional zero-bias magnetoelectric measurements at additional frequencies up to 10 kHz, a second magnetoelectric measurement setup was constructed as shown in Fig. 15. Here, an AC magnetic field (ranging from 1 to 5 Oe) was applied to the nanowire array via Helmholtz coils driven by a function generator. As before, the magnetic fields were applied perpendicular to the long axis of the nanowires. The AC field was measured by a Lakeshore Hall Probe, and the output voltage is measured via a Tektronix DPO2004 Oscilloscope. In the setup, the nanowire array substrate is placed on a stage that facilitates physical rotation of the substrate in order to explore the influence of unwanted electromagnetic induction in the output signal. The induction arises due to the finite cross-sectional area determined by the substrate electrode geometry and connecting wires.

With a voltage of 20 V_pp_ @ 10 kHz applied to the Helmholtz coils, the nanowire array is rotated 360° in 30° increments, where the rotation angle is defined as *φ*. During this rotation, the fields acting on the nanowires remain in the same direction, but the electromagnetic induction signal varies both constructively and deconstructively. The observations from this measurement are subsequently fit to a cosine wave, which is then used to determine the angle *φ*_min,ind_ at which inductive effects have approximately no contribution to the output voltage of the array. Here the angles at which induction has the maximum additive and subtractive contribution were also determined. To minimize inductive effects on the magnetoelectric measurements the substrate was positioned to the angle found above of *φ*_min,ind_ while obtaining the magnetoelectric coefficient vs frequency spectra as detailed below. Additionally to examine the maximum destructive and constructive contributions of induction, from our array and electrical connections, to the effective magnetoelectric coefficient spectra were also taken at angles *φ*_min,ind_ ± 90°.

A spectra of the zero-bias magnetoelectric coefficient at frequencies ranging from 20 Hz to 10 kHz was obtained using applied AC magnetic fields between 1 and 5 Oe. At a given frequency, the voltage output of the nanowire array and the Hall probe output were measured for 5 different voltages applied to the Helmholtz coil (6, 7, 8, 9, and 10 V_pp_). The magnetoelectric coefficient at that frequency was found by performing a linear regression to the voltage amplitude vs. field amplitude data. This process was automated via computer control of the instruments and subsequent data analysis with Python and PyVISA.
